# Multiple consecutive norovirus infections in the first 2 years of life

**DOI:** 10.1007/s00431-015-2591-8

**Published:** 2015-07-09

**Authors:** Vesna Blazevic, Maria Malm, Marjo Salminen, Sami Oikarinen, Heikki Hyöty, Riitta Veijola, Timo Vesikari

**Affiliations:** Vaccine Research Center, University of Tampere Medical School, Biokatu 10, FI-33520 Tampere, Finland; Department of Virology, University of Tampere Medical School, Tampere, Finland; Fimlab Laboratories, Pirkanmaa Hospital District, Tampere, Finland; Department of Pediatrics, Medical Research Center, University of Oulu, Oulu, Finland

**Keywords:** Norovirus, Infant, Immunity, Infections, Serology

## Abstract

Studies investigating the magnitude and breath of protective immune responses after primary and subsequent norovirus infections in pediatric populations are limited. We investigated incidence of norovirus infections and serological responses in a child from longitudinal stool and serum samples collected from birth to 2 years of age. Four consecutive infections with distinct genotypes of norovirus were detected. Serum antibodies were genotype-specific offering no protection to reinfection with heterologous virus.

*Conclusion*: This study describes norovirus-specific serological responses in a child with four consecutive norovirus infection during the first 2 years of life. The response is type-specific and does not protect from a subsequent infection with a heterologous virus.

**Table:** 

**What is Known:** • *Correlates of protection to norovirus infection and disease are not yet determined, and most of the presently available data concern adult population.*
**What is New:** • *This manuscript describes serological immune responses after primary and subsequent infections in a child during the first 2 years of life.*

## Introduction

Noroviruses (NoVs) cause great burden of acute gastroenteritis worldwide in children under 5 years of age with approximately 200,000 deaths annually [1]. The virus is genetically highly diverse with more than 30 genotypes belonging to six genogroups (GI–GVI) [2]. NoV infections in children are associated with GI and GII, with genotype GII.4 being predominant for a long period of time [2]. These infections are acquired early in life, and there is limited knowledge on protective immune responses and duration of protection in natural infection in children. A longitudinal study of NoV infection in young children has recently demonstrated that reinfections with distinct genotypes commonly happen [3,4], but the immunity was not directly measured. In a recent case report [5], NoV-specific mucosal antibodies did not protect a child from re-infection with heterologous NoV. In here, we report a child followed from birth to 2 years of age with four NoV infections and emergence of acquired NoV-specific serum IgG and blocking antibodies. NoV-specific serum antibodies, which block binding of NoV capsid-derived virus-like particles (VLPs) to the host cell attachment factors, histo-blood group antigens (HBGAs), are considered as correlates of protection to NoV infection [6].

## Materials and methods

A healthy newborn was recruited in 2001 into the prospective Type 1 Diabetes Prediction and Prevention (DIPP) Study starting at birth [7]. The study was approved by the ethics committee of the University of Oulu and Oulu University Hospital and a written informed consent was obtained from the parents. Serum samples were collected at 0 (cord blood), 3, 6, 12, 18, and 25 months of age, and stool samples were collected monthly from 4 to 19 months of age (Table 1).

**Table 1:** Detection of norovirus in stool samples and GI.3- and GII.4-specific antibodies in serum samples of a child from 0 to 25 months of age

Age (month)	Stool collection	Serum collection	RT-PCR (stool samples)	Serum IgG ELISA	
				GI.3-specific	GII.4-specific
0 (cord blood)	No	Yes		+	+
3	No	Yes		+	+
4	Yes	No	GII.6		
5	Yes	No	−		
6	Yes	Yes	−	++	++
7	Yes	No	−		
8	Yes	No	−		
10	Yes	No	−		
11	Yes	No	−		
12	No	Yes		+	+
13	Yes	No	GI.3		
14	Yes	No	−		
15	Yes	No	GII.4		
16	Yes	No	−		
17	Yes	No	−		
18	No	Yes		++	++
19	Yes	No	GII.2		
25	No	Yes		+	+

− negative, + positive, ++ indicates fourfold increase in GI.3- and GII.4-specific end-point titer

Reverse transcription-PCR (RT-PCR) and ORF1 polymerase (region A) sequencing were used for detecting NoV genotype from stool suspensions according to the well-established methods [8]. NoV GI.3 and GII.4 VLPs cloned from original patient sequences from 2002 (GI.3, GenBank reference strain accession no. AF414403) and 1999 (GII.4, AF080551) were produced in baculovirus-insect cell expression system as earlier described [9]. These VLPs were chosen as antigens to detect NoV GI- and GII-specific IgG antibody responses using an ELISA method [10] as these sequences originated near the time of serum collections in this study (2001–2003, respectively). Briefly, VLPs were coated in phosphate-buffered saline (0.5 μg/ml) on 96-well polystyrene plates (Costar, Corning, NY, USA). Twofold serial dilutions of serum samples, starting at 1:100, were incubated on blocked plates for 1 h at 37 °C. Bound antibodies of serially diluted sera were detected with goat anti-human IgG-HRP (Invitrogen, CA, USA) followed by *o*-phenylenediamine dihydrochloride (OPD) substrate (Sigma-Aldrich, MO, USA). Optical density (OD) was measured, and a mean OD ≥0.100 was considered positive. End-point titer was expressed as a reciprocal of final serum dilution having positive OD. Seroconversion was defined as at least fourfold increase in successive serum end-point titer. Serum antibodies able to block binding of GI.3 and GII.4 VLPs to HBGAs in human saliva and therefore potentially neutralize the virus were tested in blocking assay as previously described [10]. Briefly, 96-well plates were coated with type A saliva from a secretor-positive adult at 1:3000 dilution. Serially, twofold diluted sera (starting dilution 1:50) preincubated with GI.3 or GII.4 VLPs (0.1 μg/ml) for 1 h at 37 °C were added to the plates. VLPs without serum were used as maximum binding controls. Bound VLPs were detected with NoV genotype-specific mouse sera and anti-mouse IgG-HRP (Sigma-Aldrich) and OPD substrate. Blocking index (%) was calculated as 100 % − [(OD wells with VLP serum mix/OD wells without serum; maximum binding) × 100]. Blocking titer 50 (BT50) was determined as the reciprocal of the final serum dilution that blocked at least 50 % of VLPs binding to the HBGA.

## Results

**Fig. 1:**
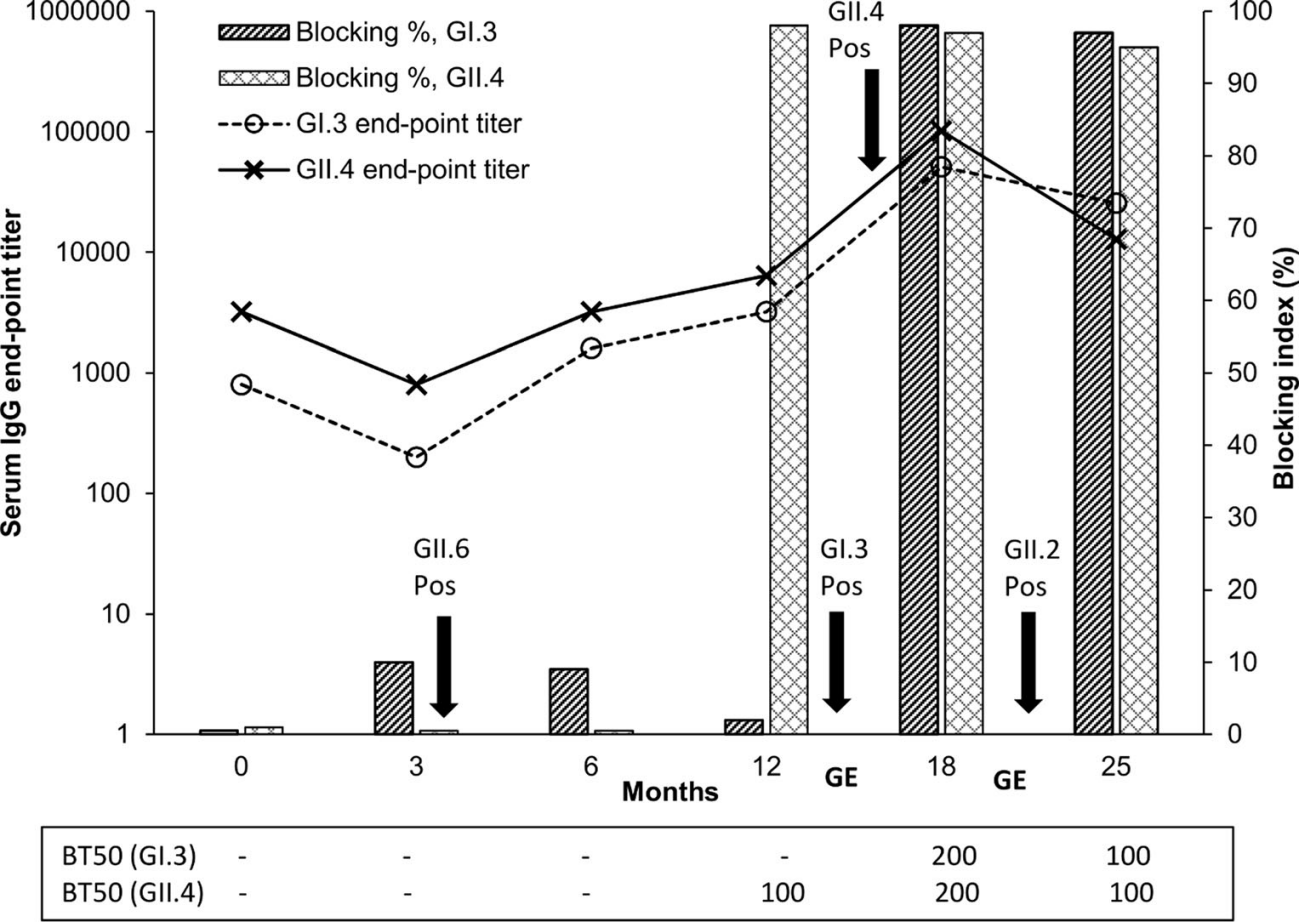
Norovirus-specific serum IgG antibodies in a child from 0 to 25 months of age. Serum samples collected at 0, 3, 6, 12, 18, and 25 months were tested for GI.3- and GII.4-specific antibodies in ELISA and blocking assays. End-point titer is expressed as a reciprocal of final serum dilution giving an OD ≥ 0.100. Blocking index (%) is shown for serum dilutions 1:50. Blocking titer 50 (BT50), reciprocal of the final serum dilution with 50 % blocking activity. Approximate time points of norovirus infections are indicated by *arrows. GE* stands for gastroenteritis symptoms reported

Four different NoV infections, three with GII (GII.6, GII.4, and GII.2) and one with GI (GI.3) NoVs, were detected by RT-PCR during a period of 15 months (Table 1). The child acquired febrile primary infection with GII.6 already at 4 months indicating that maternal antibodies although present in cord blood (end-point titer 800 to GI.3 and 3200 to GII.4, respectively) did not protect the child from NoV infection (Table 1 and Fig. 1). However, the cord blood antibodies completely lacked blocking potential of both VLPs (Fig. 1). Following the first infection, IgG seroconversion to GI.3 and GII.4 was detected at 6 months (Table 1 and Fig. 1). Although the levels of antibodies pertained until 12 months of age, they did not confer protection from a subsequent infection with GI.3 NoV at 13 months of age, and even though GII.4-specific antibodies detected at 12 month at a serum dilution 1:50 blocked 98 % of GII.4 VLPs binding to the HBGA receptor/s (Fig. 1), they did not protect the child from acquiring GII.4 infection at 15 months of age (Table 1). Clinically, the child experienced a prolonged diarrhea for a period of 3–4 weeks at the age of 13–14 months. During that period, the child might have experienced the two sequential infections with GI.3 and GII.4 NoVs. After the infections with GI.3 and GII.4, serum antibody levels to both VLPs measured at 18 months increased 16 times (end-point titers 51,200 and 102,400, respectively) compared to the pre-infection titers at 12 months (end-point titers 3200 and 6400) (Fig. 1). The high antibody level to GII.4 genotype (end-point titer 102,400 with 97 % blocking) did not prevent further infection with heterologous GII.2 NoV genotype already in the following month at the age of 19 months (Table 1 and Fig. 1), when the child again had typical clinical symptoms of gastroenteritis. These results show complete lack of cross-protection between the GII NoVs. In 7 months’ time (from 18–25 months), the child had retained only a half of GI.3 (end-point titer 25,600) and 1/8 of GII.4-specific antibodies (end-point titer 12,800) in the serum (Fig. 1).

## Discussion

Our study shows that a child can acquire as many as four consecutive NoV infections before the age of 2 years. Upon primary infection with GII.6 at 4 months, the child presented fever, but as there were no signs of vomiting or diarrhea, the infection was probably mild or asymptomatic, as recently reported [3]. Maternal antibodies present prior to the infection might have protected from severe disease but not the infection. A seroconversion to GII.4 was detected after the primary infection already at 6 months, but increase in blocking antibodies to GII.4 was detected at 12 months, suggesting that heterologous blocking antibodies may take longer time to develop. Although the child generated remarkable homologous serum antibody titer (GII.4-specific end-point titer 102,400, respectively) with high blocking activity (BT50 200), the immunity was not cross-protective and the child remained prone to infections with new GII.2 NoV genotype. The results presented here are in contrast to the findings observed in rotavirus infections, where protective antibody titers were achieved after two consecutive symptomatic or asymptomatic rotavirus infections in children less than 2 years of age [11]. The results in the present study suggest that NoV protective immunity in young children is type-specific. However, further studies in large pediatric populations are needed to address the magnitude, specificity, and duration of immune responses required for protection to NoV infection in young children. As children up to 2 years of age are highly vulnerable to infection and severe medically attended gastroenteritis, NoV vaccination of this target population is needed [12].

## Abbreviations

DIPP: Type 1 diabetes prediction and prevention
ELISA: Enzyme-linked immunosorbent assay
G: Genogroup
HBGA: Histo-blood group antigens
HRP: Horseradish peroxidase
IgG: Immunoglobulin G
NoV: Norovirus
OD: Optical density
OPD: *o*-Phenylenediamine dihydrochloride
ORF: Open reading frame
PBS: Phosphate-buffered saline
RT-PCR: Reverse transcription polymerase chain reaction
VLP: Virus-like particle
